# The mediation role of sleep quality in the relationship between cognitive decline and depression

**DOI:** 10.1186/s12877-022-02855-5

**Published:** 2022-03-03

**Authors:** Xiaolei Liu, Xin Xia, Fengjuan Hu, Qiukui Hao, Lisha Hou, Xuelian Sun, Gongchang Zhang, Jirong Yue, Birong Dong

**Affiliations:** 1 National Clinical Research Center for Geriatrics, West China Hospital, Sichuan University, No. 37, Guo Xue Xiang Renmin Nan Lu, Chengdu, Sichuan Province China; 2 Geriatric Health Care and Medical Research Center, Sichuan University, Chengdu, Sichuan Province China

**Keywords:** Cognitive decline, Depression, Mediation analysis, Sleep quality

## Abstract

**Objectives:**

Associations between cognitive decline and depression have been inconclusive. We examined 1) whether sleep quality mediates these relationships and 2) which factor of sleep quality mediates these relationships.

**Methods:**

This study utilized baseline data from the 2018 West China Health and Aging Trend study (WCHAT), a large cohort data-set that including participants aged over 50 years old. We defined depression using the 15-item Geriatric Depression Scale (GDS-15). Cognitive status was measured using the Short Portable Mental Status Questionnaire (SPMSQ) and sleep quality was assessed using the Pittsburgh sleep quality index (PSQI). Direct relationships between cognitive decline, sleep quality and depression were assessed using multiple linear regression. Mediation models and structural equation model (SEM) pathway analysis were used to test the mediating role of specific aspects of sleep (e.g., quality, duration) in the relationship between cognitive decline and depression.

**Results:**

Of 6828 participants aged 50 years old or older, the proportion of depression was 17.4%. Regression analysis indicated a total association between cognitive scores (β = 0.251, 95% CI 0.211 to 0.290, *p* < 0.001) and depression status. After adjusted PSQI scores, the association between cognitive scores and depression status was still significant (β = 0.242, 95% CI 0.203 to 0.281, *p* < 0.001), indicating a partial mediation effect of sleep quality. Mediation analysis verified sleep quality partially mediate the associations between cognitive decline and depression (indirect effect estimate = 0.0308, bootstrap 95% CI 0.023 to 0.040; direct effect estimate = 0.3124, bootstrap 95% CI 0.269 to 0.350). And daytime dysfunction had a highest mediation effect with a proportion of mediation up to 14.6%.

**Conclusions:**

Sleep quality partially mediated the relationship between cognitive decline and depression. Daytime dysfunction had a highest mediation effect. Further research is necessary to examine the effects of sleep quality on the relationship of cognitive decline and depression.

## Introduction

With an aging population, late-life depression has been a severe health problem in rural China which was a major cause of global disability and suicide, and associated with cardiovascular diseases and mortality [1]. Recent research reported the prevalence of depression was 15.5% in China in a large cross-sectional study which included 19,379 healthcare workers from 25 provinces [2]. However, in old people, the prevalence of depression was higher. It was found that 17.1% of males and 23.1% of females in 950 participants aged≥60 years from 22 locations in China were identified as having depressive symptoms [3]. Many studies have identified that behavioral and psychosocial factors, such as alcohol abuse, smoking, sleep disturbance, physical inactivity, unhealthy eating habits, and stressful events, and sociodemographic factors, such as low income, unemployment, low education level, and low social support, are mostly related with an increased risk of depression [4, 5].

Besides these risk factors, cognitive decline was found to be associated with depression in the recent years. It was found that cognitive impairment may be one of the more practically important aspects of depression [6]. And depression was also a risk factor for Alzheimer’s disease [7]. Older adults who were diagnosed for the first time with depression after 65 years of age, showed a stronger association with cognitive impairment (OR = 6.65, 95% CI 2.390 to 10.900, *p* < 0.01) [8]. What’s more, sleep quality was both related with cognitive decline and depression [9]. Approximately half of older people report sleep disturbances, which are associated with various health conditions. Specifically, components of poorer sleep quality and greater sleep disturbance were related to worse sustained attention scores, while increased sleep latency and daytime sleepiness were associated with greater frequency and seriousness of forgetting [10]. Night sleep disturbances (OR = 1.95, 95% CI 1.170 to 3.250) and daytime sleepiness (OR = 1.93, 95% CI 1.160 to 3.200) were also associated with depression [11]. Particularly, a study found that daytime sleepiness and poor efficiency were significantly associated with loss of interest; and poor satisfaction, daytime sleepiness, mid-sleep time, and efficiency were significantly associated with having at least one depressive symptom [12].

Both the quality of sleep and cognitive decline impact depression while they influence each other. However, the underlying mechanisms are not yet clear. In our study, we hypothesized that participants with depression would have poorer scores on cognitive tests and that this association would be mediated by sleep quality. This leads us to explore the mediating effect of sleep quality between cognitive decline and depression in older adults and further test the components of sleep quality in this relationship.

## Method

### Study design and sample population

Our study was a cross-sectional analysis obtaining baseline data of the west China health and aging trend (WCHAT) study which was conducted from 2018 to 2020 [13]. The research was approved by the Ethical Review Committee of West China Hospital with the committee’s reference number 2017(445) and the registration number is ChiCTR 1,800,018,895. All methods were performed in accordance with the relevant guidelines and regulations. Our method of sampling is multi-stage cluster sampling as follows and the response rate was 50.2% in the baseline data collection:

1. According to the distribution of Chinese ethnicities, select the gathering places of the major ethnic gathering provinces in the west China: Sichuan, Yunnan, Guizhou, and Xinjiang. 2. Select ethnic gathering places in each province: Chengdu (Ethnic Han), Maoxian (Ethnic Qiang), Zoige (Ethnic Tibetan), Kangding (Ethnic Tibetan), Mianning (Ethnic Yi), Zhenyuan (Ethnic Miao), Kunming (Ethnic Hui), Akto county (Ethnic Uyghur and ethnic Kirgiz), Dali (Ethnic Bai), Shiling (Ethnic Yi), Miyi (Ethnic Lisu). 3. Consider the topographical characteristics of various ethnic regions, scattered living, and choose a county with convenient transportation and concentrated living. A county medical institution was selected as survey site. 4. In the selected counties, some adjacent towns are randomly selected. 5. Local government carried out publicity in advance in villages and villagers voluntarily signed up for the project.

**Fig. 1:**
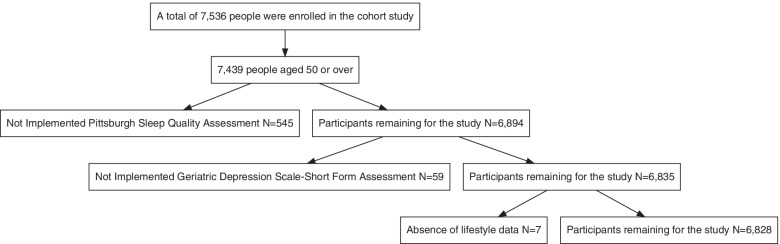
Flow chart of study participants. Initially, 7536 participants were enrolled. Then we excluded subjects who were under 50 years old and 97 participants were excluded. Then we kept on excluding 545 subjects without doing sleep quality assessment. Besides, 59 participants were excluded without doing depression assessment. After that, 7 subjects were excluded without life style data. Therefore, 6828 participants were analyzed in our study

In our study, participants aged 50 years old or older were enrolled. Participants were recruited by the local government and asked verbally by the researchers about their willingness to take part in the study. Before investigation, informed consent was signed and obtained by each participant. Initially, 7536 participants were enrolled. Then we excluded subjects who were under 50 years old and 97 participants were excluded. Then we kept on excluding 545 subjects without doing sleep quality assessment. Besides, 59 participants were excluded without doing depression assessment. After that, 7 subjects were excluded without life style data. Therefore, 6828 participants were analyzed in our study (Fig. 1).

### Data collection

We used electronic questionnaire and instruments to collect the required data. All the foreign questionnaires have been verified in China. All interviewers were medical students who were trained on collecting questionnaire data through face to face, one-on-one personal interviews. Other anthropometric and bioimpedance measurements were collected by trained technicians [13]. Depression was assessed using the 15-item Geriatric Depression Scale (GDS-15). The scale, which contains 15 items that require only a yes/no answer, is the most widely used scale for the detection of depression and scores ≥5 indicate depression [14]. Sleep quality was assessed using the Pittsburgh sleep quality index (PSQI). The questionnaire included 7 components and component scores range from 0 to 3 and a global score ranging from 0 to 21, with higher scores indicating worse sleep quality. Mostly, scores > 5 are considered as poor self-reported sleep quality [15]. Cognitive status was measured using a 10-item Short Portable Mental Status Questionnaire (SPMSQ). For SPMSQ scoring, 0 ~ 2 indicated complete cognitive function, 3 ~ 4 indicated mild cognitive functional impairment, 5 ~ 7 indicated moderate cognitive function impairment, and 8 ~ 10 indicated severe cognitive function impairment and this assessment should be based on the education level [16]. Specifically, if the education level was primary school and below, 0 to 3 errors were considered as good cognitive function; 4 to 5 errors were considered as mild cognitive decline; 6 to 8 errors were considered as moderate cognitive decline and 9 to 10 errors were considered as severe cognitive decline. If the education level was middle school, 0 to 2 errors were considered as good cognitive function; 3 to 4 errors were considered as mild cognitive decline; 5 to 7 errors were considered as moderate cognitive decline and 8 to 10 errors were considered as severe cognitive decline. If the education level was high school and above, 0 to 1 errors were considered as good cognitive function; 2 to 3 errors were considered as mild cognitive decline; 4 to 6 errors were considered as moderate cognitive decline and 7 to 10 errors were considered as severe cognitive decline [16]. The anxiety status was measured by the (GAD-7) instrument and the scores ≥5 was considered as having anxiety. Dancing was measured by asking dancing frequency and time of duration. Smoking was measured by asking smoking frequency, number of cigarettes and whether to quit smoking. Drinking alcohol was measured by asking the frequency, amount and whether to stop alcohol consumption. Drinking tea was measured by asking the frequency, type of tea and whether to stop tea drinking. These life style factors were found to be related with depression in most studies. Other baseline demographic information included age, gender, occupation, educational level, ethnic groups background. A medical history of chronic disease was self-reported. These disease conditions included hypertension, diabetes, osteoarthrosis, coronary heart disease, tumor, deafness and having two or more disease was considered as comorbidity.

### Statistical analysis

Information was processed and analyzed using R version 4.0.2. Characteristics of baseline data were presented with mean values, standard deviation (SD), and frequencies. Differences between the categories of depression and the variables studied were analyzed through analysis of variance (ANOVA) in the continuous variables and the chi squared test on categorical variables [17]. Direct effects of cognitive decline (operationalized by SPMSQ scores) and poor sleep quality (operationalized by PSQI scores) on depression were assessed using three multi-variable regression models that included relevant variables from previous covariate analyses. 95% confidence intervals were generated for all regression coefficients. Mediation hypotheses of 7 components of PSQI questionnaire and PSQI total score on the relationship between cognitive decline and depression were done using the bias-corrected bootstrap method with 6828 samples to calculate confidence intervals (95%). The results were statistically significant with *p* < 0.05. An indirect effect was considered significant when the confidence interval did not include zero. Besides, path analysis of 7 components of PSQI questionnaire was shown in the SEM framework which was done using a SEM package in R version 4.0.2 [18].

## Results

Overall, we enrolled 6828 participants (2562 men and 4266 women) aged 50 years old or older in the study. The mean age of the group was 62.43 ± 8.28 years. Table 1 shows descriptive characteristics of the participants according to depression assessment. The prevalence of depression according to the GDS-15 was 17.4%, with a higher prevalence of depression in women than in men and a lower prevalence in Han compared to other ethnic groups. Subjects with depression tended to be farmers and has a lower educational level (*p* < 0.001). It was observed that individuals in the depression group presented higher scores in subjective sleep quality, sleep latency, habitual sleep efficiency, sleep disturbance, used sleep medication, daytime dysfunction and PSQI total scores (*p* < 0.001). Subjects who enjoy dancing, drinking tea and smoking has a lower prevalence of depression (*p* < 0.001). And subjects with anxiety or cognitive decline has a higher prevalence of depression (*p* < 0.001). Besides, subjects with coronary heart disease (CHD), osteoarthrosis, deafness has a higher prevalence of depression (*p* < 0.05).

**Table 1:** Sample characteristics stratified by depressed status (*N* = 6828)

Characters	Total ***n*** = 6828	Depressed ***n*** = 1185(17.4)	Non-depressed ***n*** = 5643(82.6)	*p*
**Ethnic group, n(%)**				< 0.001
**Han**	2472(36.2)	338(28.52)	2134(37.82)	
**Others**	4356(63.8)	847(71.48)	3509(62.18)	
**Gender, n(%)**				< 0.001
**Male, n(%)**	2562(37.52)	367(30.97)	2195(38.9)	
**Female, n(%)**	4266(62.48)	818(69.03)	3448(61.1)	
**Age, mean ± SD**	62.43 ± 8.28	62.78 ± 8.50	62.36 ± 8.23	0.1245
**Age group, n(%)**				0.2511
**50–59**	2697(39.5)	454(38.31)	2243(39.75)	
**60–69**	2723(39.88)	463(39.07)	2260(40.05)	
**70–79**	1215(17.79)	235(19.83)	980(17.37)	
**80 +**	193(2.83)	33(2.78)	160(2.84)	
**Occupation**				< 0.001
**Farmers**	4456(65.26)	910(76.79)	3546(62.84)	
**Others**	2372(34.74)	275(23.21)	2097(37.16)	
**Educational level, n(%)**				< 0.001
**No formal education**	1897(27.78)	434(36.62)	1463(25.93)	
**Elementary school**	2318(33.95)	425(35.86)	1893(33.55)	
**Middle school**	1468(21.5)	200(16.88)	1268(22.47)	
**High school and above**	1145(16.75)	126(10.63)	1019(18.04)	
**Dwelling status, n(%)**				0.006
**Solitude**	339(4.96)	78(6.58)	261(4.63)	
**Non-solitude**	6489(95.04)	1107(93.42)	5382(95.37)	
**Subjective Sleep Quality, n(%)**				< 0.001
**0 score**	1243(18.2)	163(13.76)	1080(19.14)	
**1 score**	3597(52.68)	584(49.28)	3013(53.39)	
**2 score**	1688(24.72)	351(29.62)	1337(23.69)	
**3 score**	300(4.39)	87(7.34)	213(3.77)	
**Sleep Latency, n(%)**				< 0.001
**0 score**	1451(21.25)	172(14.51)	1279(22.67)	
**1 score**	2654(38.87)	425(35.86)	2229(39.5)	
**2 score**	1940(28.41)	431(36.37)	1509(26.74)	
**3 score**	783(11.47)	157(13.25)	626(11.09)	
**Sleep Duration, n(%)**				0.0563
**0 score**	3336(48.86)	550(46.41)	2786(49.37)	
**1 score**	1546(22.64)	263(22.19)	1283(22.74)	
**2 score**	1228(17.98)	225(18.99)	1003(17.77)	
**3 score**	718(10.52)	147(12.41)	571(10.12)	
**Habitual Sleep Efficiency, n(%)**				< 0.001
**0 score**	4800(70.3)	763(64.39)	4037(71.54)	
**1 score**	1041(15.25)	214(18.06)	827(14.66)	
**2 score**	425(6.22)	81(6.84)	344(6.1)	
**3 score**	562(8.23)	127(10.72)	435(7.71)	
**Sleep Disturbance, n(%)**				< 0.001
**0 score**	258(3.78)	25(2.11)	233(4.13)	
**1 score**	4422(64.76)	676(57.05)	3746(66.38)	
**2 score**	2053(30.07)	446(37.64)	1607(28.48)	
**3 score**	95(1.39)	38(3.21)	57(1.01)	
**Used Sleep Medication, n(%)**				< 0.001
**0 score**	6630(97.1)	1131(95.44)	5499(97.45)	
**1 score**	80(1.17)	14(1.18)	66(1.17)	
**2 score**	50(0.73)	15(1.27)	35(0.62)	
**3 score**	68(1)	25(2.11)	43(0.76)	
**Daytime Dysfunction, n(%)**				< 0.001
**0 score**	2815(41.23)	307(25.91)	2508(44.44)	
**1 score**	2125(31.12)	424(35.78)	1701(30.14)	
**2 score**	1392(20.39)	298(25.15)	1094(19.39)	
**3 score**	496(7.26)	156(13.16)	340(6.03)	
**PSQI**^a^**, mean(SD)** **median, (Q1-Q3)**	6.16(3.29) 6(4–8)	7.18(3.52) 7(4–9)	5.95(3.20) 5(4–8)	< 0.001
**Dancing status, n(%)**				0.0295
**Yes**	1346(19.71)	206(17.38)	1140(20.2)	
**No**	5482(80.29)	979(82.62)	4503(79.8)	
**Smoking history, n(%)**				< 0.001
**Yes**	1315(19.26)	179(15.11)	1136(20.13)	
**No**	5513(80.74)	1006(84.89)	4507(79.87)	
**Drinking alcohol history, n(%)**				0.2663
**Yes**	1315(19.26)	214(18.06)	1101(19.51)	
**No**	5513(80.74)	971(81.94)	4542(80.49)	
**Drinking tea history, n(%)**				< 0.001
**Yes**	3042(44.55)	471(39.75)	2571(45.56)	
**No**	3786(55.45)	714(60.25)	3072(54.44)	
**Anxiety status, n(%)**				< 0.001
**Yes**	1490(21.82)	530(44.73)	960(17.01)	
**No**	5338(78.18)	655(55.27)	4683(82.99)	
**Cognitive score**^a^**, mean(SD)** **median (Q1-Q3)**	1.13 ± 1.50 1(0–2)	1.64 ± 1.85 1(0–3)	1.03 ± 1.39 1(0–1)	< 0.001
**Cognitive status, n(%)**				< 0.001
**No cognitive decline**	5776(84.59)	871(73.5)	4905(86.92)	
**Mild cognitive decline**	755(11.06)	207(17.47)	548(9.71)	
**Moderate to severe cognitive decline**	297(4.35)	107(9.03)	190(3.37)	
**Hypertension, n(%)**				0.757
**Yes**	1736(25.42)	306(25.82)	1430(25.34)	
**No**	5092(74.58)	879(74.18)	4213(74.66)	
**Diabetes, n(%)**				0.8324
**Yes**	500(7.32)	89(7.51)	411(7.28)	
**No**	6328(92.68)	1096(92.49)	5232(92.72)	
**Coronary heart disease (CHD), n(%)**				0.0114
**Yes**	276(4.04)	64(5.4)	212(3.76)	
**No**	6552(95.96)	1121(94.6)	5431(96.24)	
**Osteoarthrosis, n(%)**				0.0387
**Yes**	756(11.07)	152(12.83)	604(10.7)	
**No**	6072(88.93)	1033(87.17)	5039(89.3)	
**Tumour, n(%)**				0.4499
**Yes**	49(0.72)	11(0.93)	38(0.67)	
**No**	6779(99.28)	1174(99.07)	5605(99.33)	
**Deafness, n(%)**				0.0081
**Yes**	49(0.72)	16(1.35)	33(0.58)	
**No**	6779(99.28)	1169(98.65)	5610(99.42)	
**Disease comorbidity, n(%)**				0.5049
**Yes**	1560(22.85)	280(23.63)	1280(22.68)	
**No**	5268(77.15)	905(76.37)	4363(77.32)	

Note. Means ± standard deviation was shown. Others = other nationalities including Zhuang, Manchu, Hui, Mongolia, Tujia nationalities. Data are shown using % or mean (standard deviation). *P* values were calculated with chi-squared tests and Student’s t tests for categorical and continuous variables, respectively.^a^ These variables are presented as median (interquartile range)

Table 2 showed the results of three multiple linear regression analysis in three models. Model 1 was multiple linear regression analysis between depression status and cognitive score. Model 2 was multiple linear regression analysis between depression status and cognitive scores adjusted by PSQI scores. Model 3 was multiple linear regression analysis between PSQI scores and cognitive scores. All the three models adjusted related covariates including gender, age, and ethnic group, life styles (dancing, smoking and drinking tea), chronic diseases (deafness, CHD and osteoarthrosis) and anxiety. In model 1, the results from regression analysis indicated a significant association between cognitive scores (β = 0.251, 95% CI 0.211 to 0.290, *p* < 0.001) and depression status. Model 2 showed that after adjusted PSQI scores, the association between cognitive scores and depression status was still significant (β = 0.242, 95% CI 0.203 to 0.281, *p* < 0.001), indicating a partial mediation effect of sleep quality. While model 3 showed a significant association between cognitive scores (β = 0.101, 95% CI 0.047 to 0.154, *p* < 0.001) and PSQI scores.

**Table 2:** Associations between cognitive status and sarcopenia in adults aged over 50 years old

Outcome variable	Model 1: Depressed			Model 2: Depressed			Model 3: PSQI		
	***β***	*p*	95% CI	***β***	*p*	95% CI	***β***	*p*	95% CI
**PSQI**	–	–	–	0.088	< 0.001	0.071 to 0.106	–	–	–
**Cognitive score**	0.251	< 0.001	0.211 to 0.29	0.242	< 0.001	0.203 to 0.281	0.101	< 0.001	0.047 to 0.154
**Ethnic group: Han**	− 0.236	< 0.001	− 0.353 to − 0.119	− 0.281	< 0.001	− 0.398 to − 0.165	0.509	< 0.001	0.349 to 0.669
**Gender: Female**	− 0.094	0.207	− 0.241 to 0.052	− 0.191	0.011	− 0.338 to − 0.044	1.094	< 0.001	0.893 to 1.295
**Age**	−0.002	0.568	−0.009 to 0.005	− 0.005	0.195	− 0.012 to 0.002	0.029	< 0.001	0.019 to 0.038
**Educational: Elementary school**	−0.066	0.369	−0.211 to 0.078	−0.066	0.367	−0.21 to 0.078	− 0.003	0.975	− 0.201 to 0.195
**Educational: Middle school**	−0.21	0.016	−0.381 to − 0.039	−0.193	0.026	−0.363 to − 0.023	−0.19	0.111	−0.424 to 0.044
**Educational: High school and above**	−0.563	< 0.001	−0.761 to − 0.364	−0.522	< 0.001	− 0.719 to − 0.324	−0.463	0.001	−0.735 to − 0.19
**Occupation: Farmers**	0.447	< 0.001	0.312 to 0.581	0.433	< 0.001	0.3 to 0.567	0.152	0.106	−0.032 to 0.336
**Dancing, Yes**	−0.207	0.004	−0.348 to − 0.066	−0.182	0.011	−0.322 to − 0.042	−0.289	0.003	−0.482 to − 0.096
**Smoking: Yes**	− 0.177	0.035	− 0.342 to − 0.013	−0.203	0.015	−0.367 to − 0.04	0.292	0.011	0.067 to 0.518
**Drinking tea: Yes**	−0.096	0.095	−0.208 to 0.017	−0.101	0.077	−0.213 to 0.011	0.059	0.456	−0.096 to 0.213
**Anxiety: Yes**	1.828	< 0.001	1.695 to 1.962	1.689	< 0.001	1.554 to 1.824	1.574	< 0.001	1.392 to 1.757
**Deafness: Yes**	0.561	0.084	−0.076 to 1.197	0.425	0.188	−0.208 to 1.057	1.535	0.001	0.664 to 2.406
**CHD: Yes**	0.09	0.526	−0.187 to 0.367	−0.043	0.762	−0.319 to 0.234	1.498	< 0.001	1.119 to 1.877
**Osteoarthrosis, Yes**	−0.074	0.405	−0.249 to 0.101	−0.177	0.047	−0.352 to − 0.003	1.167	< 0.001	0.928 to 1.406
**Constant**	2.309	< 0.001	1.802 to 2.817	2.06	< 0.001	1.554 to 2.566	2.826	< 0.001	2.132 to 3.519
Observations	6828			6828			6828		
R^2^	0.1675			0.1796			0.1271		
Adjusted R^2^	0.1656			0.1777			0.1251		
Residual standard error	2.249 (df = 6812)			2.233 (df = 6811)			3.077 (df = 6812)		
F Statistic (df; *P* value)	91.36 (df = (15, 6812);*P* value< 0.001)			93.22 (df = (16,6811);*P* value< 0.001)			66.1 (df = (15,6812); *P* value< 0.001)		

Note. Model 1: multiple linear regression analysis between depressed and cognitive score, Model 2: multiple linear regression analysis between depressed and cognitive score adjusted by PSQI, Model 3: multiple linear regression analysis between PSQI and cognitive score

Adjusted by gender, age, and ethnic group, life styles (dancing, smoking and drinking tea), chronic diseases (deafness, CHD, osteoarthrosis) and anxiety

**Fig. 2:**
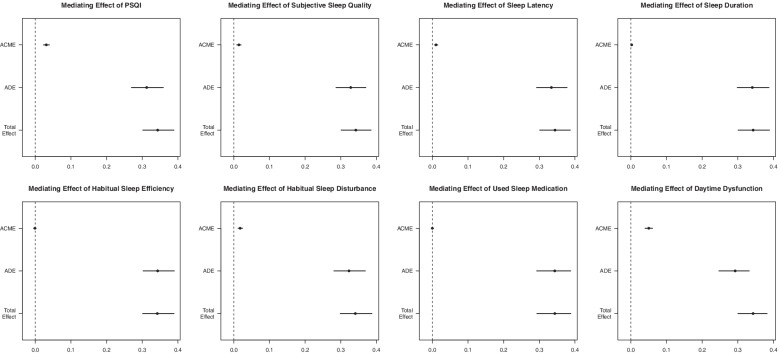
Mediation effects of PQSI and 7 sleeping elements in the relationship between cognitve decline and depression. The mediation effects are as following: PSQI (ACME = 0.0308, 95%CI 0.0231 to 0.04), subjective sleep quality (ACME = 0.0145, 95% CI 0.009 to 0.020), sleep latency (ACME = 0.010, 95% CI 0.006 to 0.020), sleep duration (ACME = 0.0026, 95% CI 0.005 to 0.010), habitual Sleep Efficiency (ACME = -0.0007, 95%CI − 0.0039 to 0), sleep disturbance (ACME = 0.018, 95% CI 0.012 to 0.020), used sleep medication (ACME = 0.0002, 95%CI − 0.0033 to 0) and daytime dysfunction (ACME = 0.0503, 95% CI 0.040 to 0.060)

Table 3 showed the relative total, direct and indirect effects for the mediating role of sleep quality on the relationship between cognitive decline and depression in mediation models. Our mediation hypothesis was confirmed because bootstrapping revealed significant relative indirect effects for depression (ACME = 0.0308, 95% CI 0.023 to 0.040), indicating that sleep quality mediated the association between cognitive decline and depression. And most of sleeping components also has mediation effect like subjective sleep quality (ACME = 0.0145, 95% CI 0.009 to 0.020), sleep latency (ACME = 0.010, 95% CI 0.006 to 0.020), sleep duration (ACME = 0.0026, 95% CI 0.005 to 0.010), sleep disturbance (ACME = 0.018, 95% CI 0.012 to 0.020) and daytime dysfunction (ACME = 0.0503, 95% CI 0.040 to 0.060). Among these components, daytime dysfunction had a highest mediation effect with a proportion of mediation up to 14.56%. And these mediation effects were also shown in Fig. 2.

**Table 3:** Mediation models: relative total, direct and indirect effects for the mediating role of sleeping quality on the relationship between cognitive decline and depression

Mediator Variable		*β*	*P*-value	95% CI
PSQI	ACME	0.0308	< 0.001	0.0231 to 0.04
	ADE	0.3124	< 0.001	0.2692 to 0.35
	Total Effect	0.3432	< 0.001	0.2976 to 0.39
	Prop. Mediated	0.0885	< 0.001	0.0686 to 0.12
Subjective Sleep Quality	ACME	0.0145	< 0.001	0.0092 to 0.02
	ADE	0.3281	< 0.001	0.2873 to 0.37
	Total Effect	0.3426	< 0.001	0.3025 to 0.39
	Prop. Mediated	0.0419	< 0.001	0.0274 to 0.06
Sleep Latency	ACME	0.0103	< 0.001	0.0061 to 0.02
	ADE	0.3331	< 0.001	0.2913 to 0.37
	Total Effect	0.3435	< 0.001	0.3028 to 0.38
	Prop. Mediated	0.0296	< 0.001	0.0173 to 0.05
Sleep Duration	ACME	0.0026	0.008	0.0005 to 0.01
	ADE	0.3408	< 0.001	0.2964 to 0.38
	Total Effect	0.3433	< 0.001	0.3016 to 0.39
	Prop. Mediated	0.0071	0.008	0.0015 to 0.02
Habitual Sleep Efficiency	ACME	−0.0007	0.68	−0.0039 to 0
	ADE	0.3443	< 0.001	0.2985 to 0.39
	Total Effect	0.3436	< 0.001	0.2974 to 0.39
	Prop. Mediated	−0.0024	0.68	−0.0117 to 0.01
Sleep Disturbance	ACME	0.018	< 0.001	0.0123 to 0.02
	ADE	0.325	< 0.001	0.2803 to 0.37
	Total Effect	0.343	< 0.001	0.297 to 0.39
	Prop. Mediated	0.0518	< 0.001	0.0357 to 0.07
Used Sleep Medication	ACME	0.0002	0.91	−0.0033 to 0
	ADE	0.3445	< 0.001	0.3006 to 0.38
	Total Effect	0.3446	< 0.001	0.3015 to 0.38
	Prop. Mediated	0.0006	0.91	−0.0101 to 0.01
Daytime Dysfunction	ACME	0.0503	< 0.001	0.0403 to 0.06
	ADE	0.2924	< 0.001	0.2519 to 0.34
	Total Effect	0.3428	< 0.001	0.3035 to 0.39
	Prop. Mediated	0.1456	< 0.001	0.1162 to 0.18

Note. ACME, average causal mediation effects (indirect effect); ADE, average direct effects; Prop. Mediated, the mediator variable explains the percentage of the association between cognitive and depressed

**Fig. 3:**
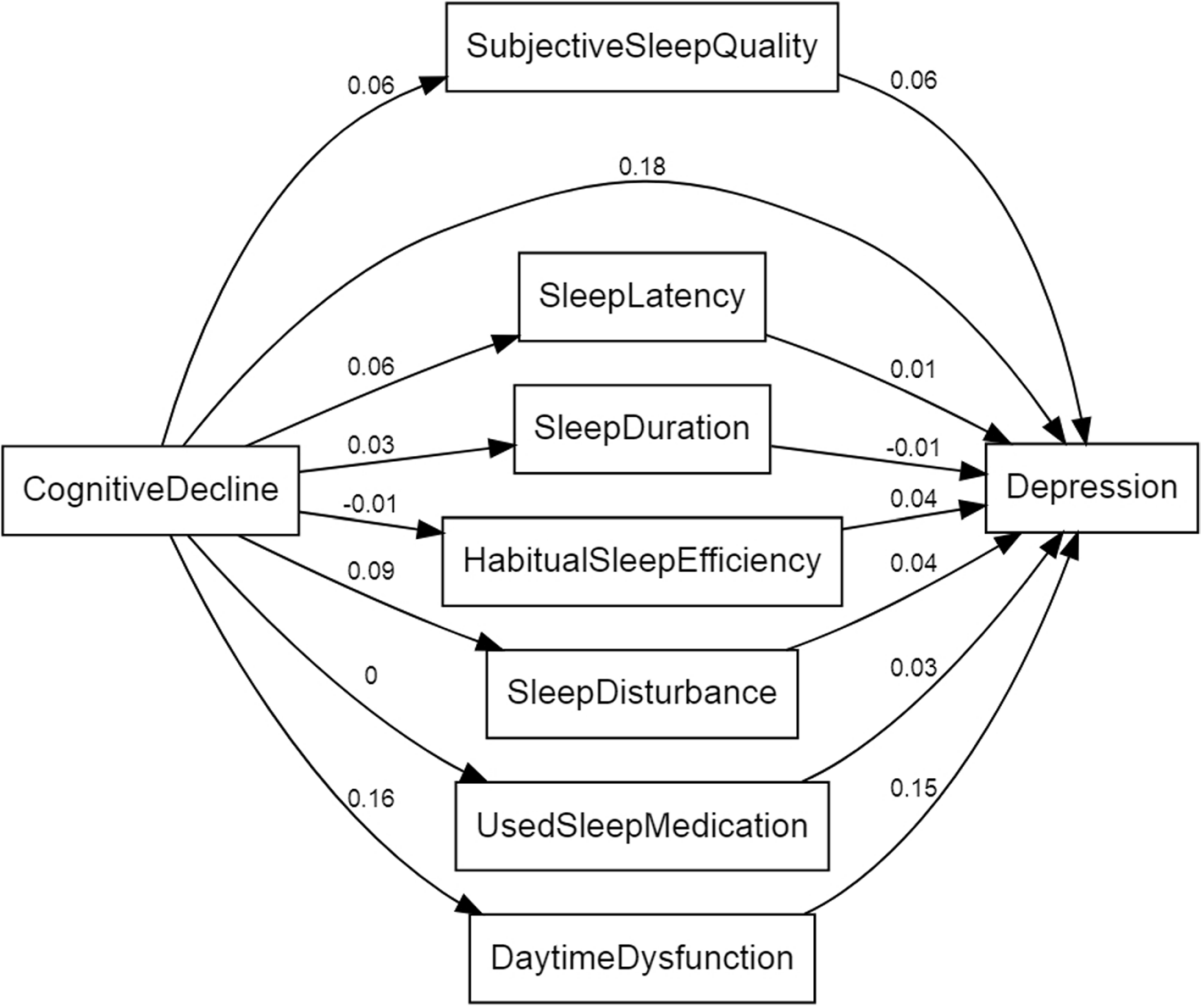
Path analysis of the sleeping quality’s mediation effects using the structural equation model (SEM) framework. SEM pathway analysis showed that the correlation between cognitive decline and depression was positive (SEM co-efficient: 0.18). 7 components of PSQI assessment were also shown in Fig. 3

We then performed path analysis using the structural equation model (SEM) framework(Chi-square statistic = 1736.2, GFI = 0.939, TLI = 0.688, RMSEA = 0.098). As shown in Fig. 3, SEM pathway analysis showed that the correlation between cognitive decline and depression was positive (SEM co-efficient: 0.18). 7 components of PSQI assessment were also shown different correlation between cognitive decline and depression. Most correlation was positive, while only the correlation between sleep duration and depression was negative (SEM co-efficient: − 0.01) and the correlation between cognitive decline and habitual sleep efficiency was negative (SEM co-efficient: − 0.01). These results further confirmed the association between cognitive decline, sleep quality and depression.

## Discussion

The current study evaluated the mediating role of sleep quality in the relationship between cognitive decline and depression. Several mechanisms have been proposed to explain how sleep quality impacts both cognition and depression. 60 to 70% of people with cognitive impairment or dementia have sleep disturbances. Research has shown that poor sleep quality as measured by the PSQI is associated with multiple markers of metabolic dysfunction, including insulin resistance which is related with bad performance on executive function tasks among older adults [19, 20]. Furthermore, several mouse models have demonstrated strong relationships between diet induced insulin resistance and memory dysfunction [21, 22]. Besides, good sleep plays a protective role in human emotional homeostasis and regulation [23]. And in depressed individuals, dysregulated sleep was often-reported [24]. As many as 90% of patients with depression will have sleep quality complaints [25]. It was reported that as many as 24 to 58% of individuals with sleep disordered breathing (eg, obstructive sleep apnea) meet the criteria for depression [26]. And it was found that among all the symptoms of depression, sleep problems are the most common (13.6%). Compared to those without sleep problems, people with sleep problems have the highest relative odds (7.6 times) of having a new major depressive episode next year. Thus, sleep disturbance was associated with having more depressive symptoms [27]. Another mechanism through which poor sleep could affect both cognitive function and depression is through oxidative stress. Higher levels of oxidative stress biomarkers were found in patients with bad sleep quality [28]. In addition, sleep deprivation is related with an increased rate of oxidative pentose phosphate pathway activity [29]. High levels of oxidative stress has also been implicated in depression among older adults [30] as well as those with cognitive decline [31]. Our study demonstrates that bad sleep quality partially mediates the effects of cognitive decline on depression in older adults. Our results thus contribute to the current knowledge by providing evidence that improving sleep quality may ameliorate the negative impact of cognitive decline on depression.

Among the 7 components of sleeping assessment, we found that daytime dysfunction had a highest mediation effect with a proportion of mediation up to 14.56%, the following was sleep disturbance which had a mediation effect of 5.18% and subjective sleep quality which had a mediation effect of 4.19%. This was consistent with previous studies. A recent research found that the effects of sleep disturbance, subjective sleep quality and daytime dysfunction scores were most obvious on anxiety in the elderly aged 60 years and older in China, and the ORs (95%CI) were 4.63 (3.55–6.04), 2.75 (2.33–3.23) and 2.50 (2.19–2.86), respectively [32]. An earlier study also found that symptoms of short sleep duration, daytime sleepiness and sleep disturbances are independently related to anxiety while the use of sleep medication is independently associated to depression in a random sample of 2393 individuals aged 65 years or older [33]. Another longitudinal study found that short sleep duration, especially on weekdays, was significantly associated with subsequent depressive (OR = 0.86, 95%CI 0.80 to 0.92) [34]. Besides, shorter sleep duration has been found to be associated with a greater rate of ventricular enlargement, which similarly reflects loss of brain volume [35]. And sleep disturbances were studied to be linked to cortical thinning, a marker of cortical atrophy found in many dementia subtypes [36, 37]. As we discussed before, numerous studies provide findings indicating the remarkable relationship between sleep alterations and depression. Our study found the most three relevant components (eg. daytime dysfunction, sleep disturbance, subjective sleep quality) mediated the relationship between cognitive decline and depression, which might be the target to focus on improving sleep quality.

According to the World Health Organization, depression is the leading cause of disability, affecting over 300 million people. Depression is also the commonest mental disorder in older adults worldwide, affecting 7% of the world’s older population and accounting for 5.7% of years lived with disability among adults aged over 60 years [38]. For many individuals with depression, the major impairment they experience is cognitive decline [39]. Our study found a high prevalence of depression that was 17.4% and after adjusting numerous confounders, the association between cognitive decline and depression was still significant. This is most likely regulated by several mechanisms. Firstly, depression’s duration has a significant impact on left hippocampal volume, indicating that the time since first depressive episode plays an important role in hippocampal degeneration which leading to cognitive decline [40]. And lower hippocampal volumes are associated with a poorer clinical outcome and more depressive episodes [41]. Secondly, accumulated evidence highlighting the major role of systemic inflammation, which were existed both in cognitive decline and depression [42]. Thirdly, as we discussed before, oxidative stress was a common mechanism in cognitive decline and depression [30, 31]. Thus, the relationship between cognitive decline and depression is complex and bidirectional. The ultimate goal of treatment in depression is fully functional recovery, and assessing patients for cognitive impairment and selecting treatments that address cognitive dysfunction.

There are several limitations in this study. Firstly, our sampling did not cover all the cities in west China. Secondly, our study design was a cross-sectional study. Thirdly, we conducted a centralized investigation and not a household survey. Furthermore, most of the participants who came to the site of investigation on their own were relatively healthy. Some bias were existed in the analysis. A critical next step would be to replicate this study with longitudinal data to establish the relationship. In addition, it would be crucial to examine if clinically established sleep interventions are able to prevent or reverse depression in cognitive decline adults.

## Conclusions

In conclusion, our study demonstrated that the relationship between cognitive decline and depression was partially mediated by sleep quality. However, our study did not find that improving sleep quality in older adults with cognitive decline could counteract the progression of depression. Further research is necessary to examine the effects of sleep quality on the relationship of cognitive decline and depression.

## Data Availability

The data-set generated and analyzed during the current study will be available two years later and is also available now from the corresponding author on a reasonable request.

## Abbreviations

WCHAT: West China Health and Aging Trend study
GDS-15: 15-item Geriatric Depression Scale
SPMSQ: Short Portable Mental Status Questionnaire
PSQI: Pittsburgh sleep quality index
SEM: Structural equation model
SD: Standard deviation
ACME: Average causal mediation effects (indirect effect)
ADE: Average direct effects
